# Activated CX3CL1/Smad2 Signals Prevent Neuronal Loss and Alzheimer's Tau Pathology-Mediated Cognitive Dysfunction

**DOI:** 10.1523/JNEUROSCI.1333-19.2019

**Published:** 2020-01-29

**Authors:** Qingyuan Fan, Wanxia He, Manoshi Gayen, Marc Robert Benoit, Xiaoyang Luo, Xiangyou Hu, Riqiang Yan

**Affiliations:** ^1^Department of Neurosciences, Lerner Research Institute, Cleveland Clinic Foundation, Cleveland, Ohio 44195, and; ^2^Department of Neuroscience, University of Connecticut Health, Farmington, Connecticut 06032

**Keywords:** Alzheimer's disease, CX3CL1, fractalkine, neurofibrillary tangles, neurogenesis, Smad2

## Abstract

Neurofibrillary tangles likely cause neurodegeneration in Alzheimer's disease (AD). We demonstrate that the CX3CL1 C-terminal domain can upregulate neurogenesis, which may ameliorate neurodegeneration. Here we generated transgenic (Tg-CX3CL1) mice by overexpressing CX3CL1 in neurons. Tg-CX3CL1 mice exhibit enhanced neurogenesis in both subgranular and subventricular zones. This enhanced neurogenesis correlates well with elevated expression of TGF-β2 and TGF-β3, and activation of their downstream signaling molecule Smad2. Intriguingly, the enhanced adult neurogenesis was mitigated when Smad2 expression was deleted in neurons, supporting a role for the CX3CL1–TGF-β2/3–Smad2 pathway in the control of adult neurogenesis. When Tg-CX3CL1 mice were crossed with Alzheimer's PS19 mice, which overexpress a tau P301S mutation and exhibit age-dependent neurofibrillary tangles and neurodegeneration, overexpressed CX3CL1 in both male and female mice was sufficient to rescue the neurodegeneration, increase survival time, and improve cognitive function. Hence, we provide *in vivo* evidence that CX3CL1 is a strong activator of adult neurogenesis, and that it reduces neuronal loss and improves cognitive function in AD.

**SIGNIFICANCE STATEMENT** This study will be the first to demonstrate that enhanced neurogenesis by overexpressed CX3CL1 is mitigated by disruption of Smad2 signaling and is independent of its interaction with CX3CR1. Overexpression of CX3CL1 lengthens the life span of PS19 tau mice by enhancing adult neurogenesis while having minimal effect on tau pathology. Enhancing neuronal CX3CL1, mainly the C-terminal fragment, is a therapeutic strategy for blocking or reversing neuronal loss in Alzheimer's disease or related neurodegenerative disease patients.

## Introduction

CX3CL1 is a membrane-anchored signaling chemokine (Bazan et al., 1997; Pan et al., 1997), which is recognized to exert its signaling function through interaction with its sole cognate receptor CX3CR1 (Imai et al., 1997). CX3CL1 is shed near the transmembrane domain to release the C-XXX-C motif containing N-terminal domain for the binding to CX3CR1. In the brain, CX3CL1 is mainly and constitutively expressed by neurons, while CX3CR1 is predominantly expressed by microglia (Hatori et al., 2002). This neuron–glia cross talk has been shown to regulate or modulate important functions such as microglial migration and activation, neuronal protection, or impairment depending on normal or diseased conditions (Limatola and Ransohoff, 2014; Paolicelli et al., 2014; Lauro et al., 2015). Recently, the short intracellular domain of CX3CL1 has been shown to mediate a back-signaling function independent of CX3CR1; Tg-CX3CL1-ct mice, which overexpress only this C-terminal domain, exhibit enhanced neurogenesis (Fan et al., 2019).

The role of CX3CL1 in Alzheimer's disease (AD) pathogenesis remains to be understood (Guedes et al., 2018). AD is characterized by amyloid deposition due to excessive accumulation of β-amyloid (Aβ) peptides, neurofibrillary tangles due to hyperphosphorylation of tau protein, neuronal loss, and synaptic dysfunction in the brain (Braak and Braak, 1997). Altered CX3CL1/CX3CR1 signaling activity likely regulates pathological changes in various ways. Deficiency in CX3CR1 may reduce amyloid deposition in AD mouse models overexpressing mutant APP or APP/PS1 (Lee et al., 2010) and prevents neuronal loss in a 3XTg AD mouse model overexpressing mutant APP/PS1/tau (Fuhrmann et al., 2010). Mice deficient in CX3CL1, the ligand of CX3CR1, were also shown to have reduced Aβ deposition (Lee et al., 2014). Others noted that decreasing CX3CL1/CX3CR1 signaling activity exacerbated plaque-independent cognitive deficits in APP-overexpressing AD mouse models (Cho et al., 2011); deficiency in CX3CR1 was found to increase tau hyperphosphorylation (Bhaskar et al., 2010; Cho et al., 2011; Lee et al., 2014) and to increase neurodegeneration (Cardona et al., 2006). Overexpressed soluble CX3CL1 via adenoviral transformation in a Tg4510 model of tauopathy reduces tau pathology and prevents neurodegeneration, but shows no effect on amyloid deposition in mice overexpressing mutant APP and PS1 (Nash et al., 2013). The postulated mechanisms include the CX3CR1-mediated microglial migration and activation, and altered release of proinflammatory cytokines such as IL-1β and TNFα (Fuhrmann et al., 2010; Lee et al., 2014). Intriguingly, overexpressing only CX3CL1-ct in 5xFAD mice can reduce amyloid deposition and neuronal loss (Fan et al., 2019), which is independent of CX3CR1 function. Hence, the N- and C-terminal fragments of CX3CL1 can impact AD pathogenesis by acting on different pathways.

To further understand the *in vivo* roles of CX3CL1, we generated transgenic Tg-CX3CL1 mice, which overexpress CX3CL1 under the control of a prion promoter, and examined the *in vivo* roles of overexpressed neuronal CX3CL1. We observed overexpressed neuronal CX3CL1 did not obviously elicit changes in inflammatory responses in Tg-CX3CL1 mice based on the morphology of microglia compared with wild-type (WT) littermates. Instead, Tg-CX3CL1 mice exhibited enhanced neurogenesis in both the subventricular zone (SVZ) and the subgranular zone (SGZ). TGF-β2/3 expression was elevated and phosphorylation of Smad2 was increased in Tg-CX3CL1 mouse brains, similar to mice overexpressing only the neuronal CX3CL1 C-terminal fragment (Fan et al., 2019). If the *smad2* gene was ablated in forebrain neurons by conditional deletion, enhanced neurogenesis in Tg-CX3CL1 was mitigated. Importantly, enhanced expression of CX3CL1 in Alzheimer's transgenic PS19 mice caused a reduction in neurodegeneration, improved cognitive function, and increased life span. Together, our results reveal that increased neurogenesis by more neuronal CX3CL1 is sufficient to reverse neuronal loss in AD.

## Materials and Methods

### 

#### 

##### Generation of Tg-CX3CL1 mice.

Tg-CX3CL1 was generated by the insertion of HA-tagged CX3CL1 human cDNA between exon 2 and exon 3 of mouse prion protein gene DNA at two unique XhoI sites in the Mo-Prp plasmid vector, and the prion promoter predominantly drives the expression of transgene in neurons (Borchelt et al., 1997). The pair of primers for the transgene used for PCR-based genotyping was 5′-ACCTGTAGCTTTGC-3′ and 5′-TTCAGACGGAGCAT-3′. Mouse RTN3-specific primers (5′-CACAGGTAGAAATGGCCAAGA-3′ and 5′-CAGCTTGAATGACAGACTTATAGACT-3′) were included in the PCR to identify mouse sequence. The identified and selected founder line was crossed with C57BL/6J mice for at least six generations to obtain a relatively pure genetic background. All experimental protocols were approved by the Institutional Animal Care and Use Committee of the Lerner Research Institute in compliance with the guidelines established by the Public Health Service *Guide for the Care and Use of Laboratory Animals*. For animal studies as discussed in each figure, both male and female mice were used and usually in relatively balanced numbers (Table 1, summary).

**Table 1.** Mice used for different experiments are summarized

Figure	Technique	Age of animals
1*C*	IHC	P30 and 11 months
2*A*,*B*	Two pulses of BrdU every 12 h followed by IHC	P21
2*C*,*D*	BrdU daily for 5 d followed by 4 weeks then IHC	P11 to P45
2*E*,*F*	Two pulses of BrdU every 12 h followed by IHC	8 months
3*A*	Brain lysates	P30
4*A*	Brain lysates	P30
4*B*	Two pulses of BrdU every 12 h followed by IHC	P21
5*A*	Brain lysates	7 months
5*B*	IHC	8 months
5*C*,*D*	IHC	11 months
6*A*	Survival curves	Up to 18 months
6*B*	IHC	11 months
7*A–C*	Behavioral assay	8 months
S1	IHC	2, 6, 11, and 20 months
S2	IHC	P11, P20, P30, and 11 months

IHC, Immunohistochemistry. S1 refers to Supplementary Figure S1. S2 refers to Supplementary Figure S2.

##### Western blotting and antibodies.

Total proteins were extracted from freshly dissected mouse tissue using modified RIPA buffer. Equal amounts of protein (30 or 50 μg) were resolved on an Invitrogen NuPAGE Bis-Tris gel (Thermo Fisher Scientific) and transferred onto an Invitrogen nitrocellulose membrane (Thermo Fisher Scientific). Routinely, at least two mice from each genotype group were used for Western blot analysis. Antibodies used for Western blotting are as follows: p-Smad2 (phosphorylated-Smad2; catalog #3104; RRID:AB_390732), Smad2/3 (catalog #8685; RRID:AB_10889933), p-Smad1 (catalog #9553s; RRID:AB_2107775), Smad1 (catalog #6944; RRID:AB_10858882), and p-tau-PHF13 (catalog #9632s, Cell Signaling Technology; RRID:AB_2266237); Calretinin (catalog #SC-11644; RRID:AB_634545), TGF-β1 (catalog #SC-146; RRID:AB_632486), TGF-β2 (catalog #SC-90; RRID:AB_2303237), TGF-β3 (catalog #SC-82; RRID:AB_2202303), and SNAP25 (catalog #SC-20038, Santa Cruz Biotechnology; RRID:AB_628264); synaptophysin mouse antibody (catalog #S5768, Sigma-Aldrich; RRID:AB_477523); p-Tau AT8 (catalog #MN1020; Thermo Fisher Scientific; RRID:AB_223647); Tau-PHF (catalog #AT-180, Thr 231, MN1040, Thermo Fisher Scientific; RRID:AB_223649); actin (catalog #A5441, Sigma-Aldrich; RRID:AB_476744); BrdU (catalog #ab6326, Abcam; RRID:AB_305426); NeuN (catalog #MAB377, EMD Millipore; RRID:AB_2298772); HA (catalog #11867431001, Roche; RRID:AB_390919); Iba1 (1:500; Wako Chemicals; RRID:AB_839504); and SMI22 (1:1000, Covance; RRID:AB_2313859). HRP-conjugated secondary antibodies were used and visualized using enhanced chemiluminescence (Thermo Fisher Scientific).

##### Immunohistochemical staining.

Immunohistochemical staining experiments were performed according to standard methods previously described (Hu et al., 2018). Routinely, half mouse brains were surgically removed, fixed in 4% paraformaldehyde (PFA) for 12 h, and immersed in 20% sucrose overnight at 4°C. Brains were sagittally sectioned (16 μm thick) on a freezing microtome (Microm). Sections were permeabilized with 0.3% Triton X-100 for 30 min. After being rinsed in PBS three times to remove detergent, the sections were heated by microwave in 0.05 m citrate-buffered saline, pH 6.0, for 5 min, blocked with 5% normal goat serum, and incubated with individual primary antibodies at the following dilutions: AT8 or AT180, 1:1000; Iba1, 1:500; SMI22, 1:1000; and synaptophysin, 1:400. After washing with PBS three times, sections were incubated with secondary antibodies conjugated with Invitrogen Alexa Fluor 488 or Alexa Fluor 568 (Thermo Fisher Scientific).

##### Neurogenesis assays.

Neurogenesis was assessed by BrdU labeling experiments as described previously (Hu et al., 2013). Briefly, mice were injected intraperitoneally with 50 mg/kg BrdU (catalog #B9285, Sigma-Aldrich) at postnatal day 21 (P21) twice at 12 h intervals. The animals were then killed 24 h after the first injection of BrdU. Brain samples were fixed in 4% PFA at 4°C overnight and then transferred into 30% sucrose in PBS for 2 d. Sections were pretreated with serial 1 m and 2 m HCl, stained with primary antibody BrdU, and then processed by using VECTASTAIN-ABC Kits (Vector Laboratories) and SIGMA*FAST* DAB with metal enhancer tablets (Sigma-Aldrich) according to the manufacturer instructions. After color development, the sections were dehydrated by incubation for 3 min in each of 70, 95, and 100% ethanol, followed by 5 min in two changes of xylenes and mounted using Permount. Labeled cells were quantified by stereology. Every third section (of a total of 30 sections) was counted under a 100× objective, and the sum was multiplied by 3 to estimate the total number of BrdU^+^ cells in the region. Cells were counted if they were in or touching the SGZ, and cells were excluded if they were more than two cell diameters from the GCL.

To analyze neural differentiation, 15 mg/kg BrdU was injected once daily for 5 d beginning at P11. The animals were killed after an additional 21 or 28 d as specified in the text, and brain sections were examined with double labeling by using primary antibodies to BrdU (1:100) and NeuN (1:1000).

##### Y-maze and novel object recognition tests.

Both behavioral tests were routinely performed in the laboratory, and all procedures were previously described in detail (Luo et al., 2014). Animals at the age of 8 months were used in this study, and the number of animals is specified in each figure. Y-maze tests were conducted first, and object recognition tests were conducted a week later.

##### Contextual fear-conditioning test.

Contextual fear-conditioning tests are conducted routinely in the Yan laboratory (Luo et al., 2014; Hu et al., 2018). The first day involves testing mice for the conditioning by placing them in the chamber (Med Associates) for 3 min (phase A) before the stimulating sound at 2800 Hz and 85 dB for 30 s (phase B, conditioning stimulus). The last 2 s was given a 0.7 mA continuous footshock (phase C, unconditioned stimulus). Phases B and C were repeated once after mice rested for 30 s in the chamber. On the second day, mice were tested for their contextual memory in the same chamber for 3 min without either sound or footshock. On the third day, mice were tested for their tone memory in a different chamber environment with the sound, but no footshock. Fear memory of the mice was measured as the percentage of freezing, which was defined as the percentage of time completely lacking movement, except for respiration, in intervals of 5 s.

##### Statistical analysis.

Quantitative data are presented as the mean ± SEM. All experiments were independently repeated at least three times. Statistical analyses were conducted using Prism 6 software (GraphPad Software). Statistical comparisons between groups were analyzed for significance by one-way ANOVA with Tukey's *post hoc* test and Student's *t* test. In the case of multiple variances, two-way ANOVA with *post hoc* Bonferroni's test and three-way ANOVA with Tukey's multiple-comparisons test were also performed. Significant *p* values are denoted by the use of asterisks in the text and figures (*p < 0.05, **p < 0.01, ***p < 0.001). Error bars in each case represent the SEM.

## Results

### Transgenic mice overexpressing CX3CL1 show enhanced neurogenesis

**Figure 1.**
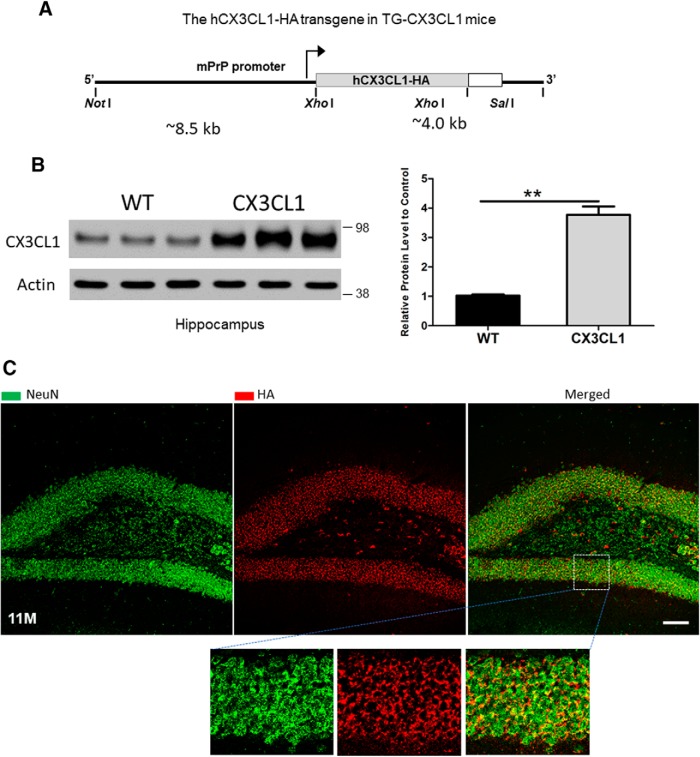
Generation of transgenic mice overexpressing CX3CL1. ***A***, Schematic illustration of the construct used for expressing CX3CL1 transgene driven by the prion promoter. Human CX3CL1 cDNA (373 aa) contains one copy of an HA tag at its C-terminal end. ***B***, Tg-CX3CL1 mice express about threefold more CX3CL1 compared with WT mice. ***C***, CX3CL1 transgene is expressed by neurons. As shown in the example of 11-month-old Tg-CX3CL1 brains, CX3CL1-HA is colocalized with NeuN, and this colocalization was clearer in the enlarged images. Tg-CX3CL1 mice cause no visible morphological changes in microglia (Figure 1-1) and astrocytes (Figure 1-2). An antibody specific to the HA tag was used for staining the transgene. Scale bar, 30 μm.

To explore the *in vivo* role of neuronal CX3CL1, we generated transgenic Tg-CX3CL1 mice, which were engineered to overexpress CX3CL1 transgene, including the heavily glycosylated mucin-like stalk of fractalkine (Fong et al., 2000), in neurons of broad brain regions by the murine prion promoter (Fig. 1*A*). After pronuclear microinjection of the linearized CX3CL1 transgene, which contains a C-terminal HA tag, 13 independent founder lines of mice were detected to express different levels of CX3CL1, and 4 lines were chosen for further characterizations. In this study, the line with approximately threefold greater expression of CX3CL1 than WT was used (Fig. 1*B*). As expected, the CX3CL1-HA transgene, detected by an antibody to HA, was predominantly expressed in neurons and was not readily detectable in glia at young and older ages (Fig. 1*C*).

10.1523/JNEUROSCI.1333-19.2019.f1-1 Figure 1-1 **Tg-CX3CL1 mice show no obvious changes in microglia**. Fixed brain sections from different age groups of Tg-CX3CL1 mice and their littermates were reacted with antibody Iba1 to label microglia. From 2-, 6-, 11- and 22-month-old samples, no obvious increase in the total number of microglia was observed, and no ramification of microglia or morphological changes were seen in Tg-CX3CL1 brains, indicating no microglial proliferation or activation was caused by overexpression of neuronal CX3CL1. Scale bar is 30 μm. Download Figure 1-1, TIF file

**Figure 2.**
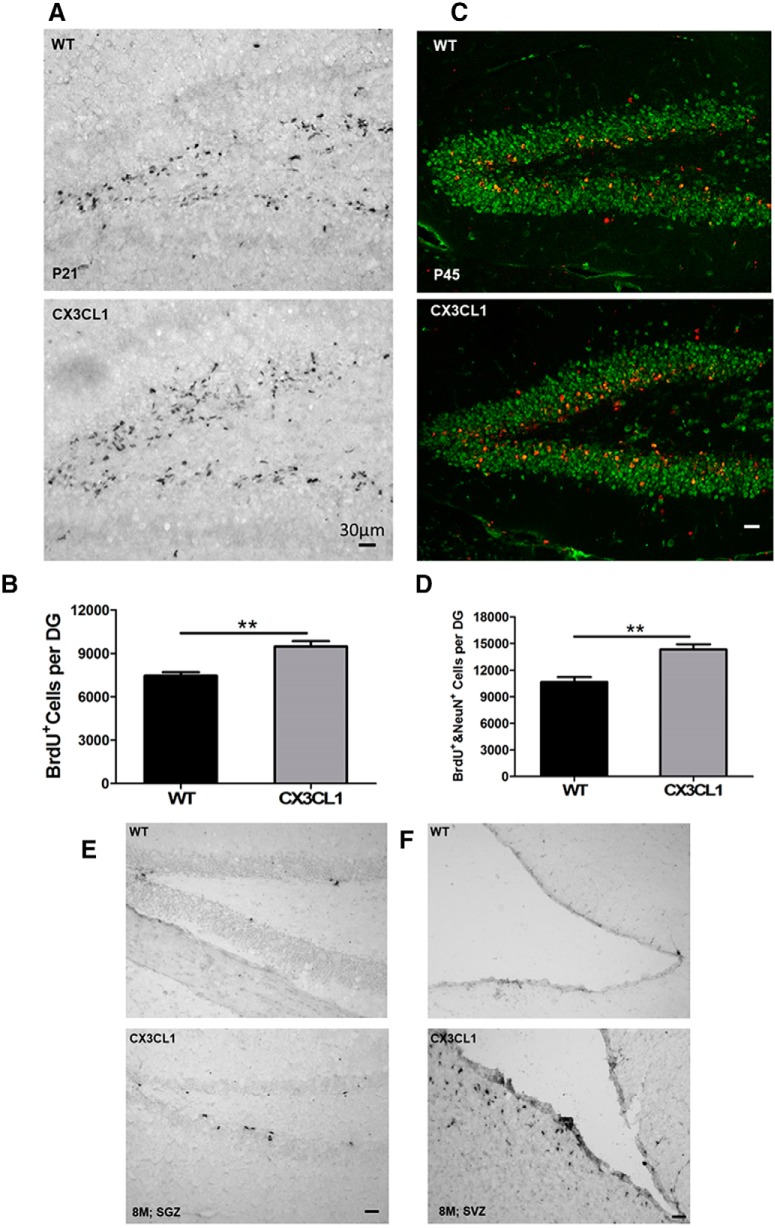
Enhanced neurogenesis following overexpression of CX3CL1. ***A***, P21 mice were BrdU pulse labeled for 24 h, and fixed brain sections were labeled by antibody to BrdU. The number of BrdU^+^ cells was significantly larger in Tg-CX3CL1 SGZ compared with that in wild-type controls. ***B***, Stereological quantification confirmed greater numbers of BrdU^+^ dividing cells in P21 Tg-CX3CL1 SGZ. *N* = 5 pairs of mice (**p < 0.01, Student's *t* test). ***C***, Mice at P45 were pulse labeled by BrdU and examined by confocal staining with antibody to BrdU (red) and NeuN (green) for mature neurons. ***D***, BrdU^+^ cells from P45 mice were counted on 1 of every 10 continuous sections, and the number reflected an average per section. *N* = 5 pairs of mice (*p < 0.05). ***E***, ***F***, Eight-month-old mice were similarly BrdU pulse labeled and examined by immunohistochemical staining with antibody to BrdU. Visibly more BrdU^+^ cells were found in both Tg-CX3CL1 SGZ (***E***) and SVZ (***F***). Scale bar, 30 μm.

10.1523/JNEUROSCI.1333-19.2019.f1-2 Figure 1-2 **No obvious changes in astrocytosis or astrogenesis are induced by neuronal CX3CL1 overexpression**. (A) Fixed brain samples were stained with Smi22 antibody for GFAP to label astrocytes. No significant increase in the numbers of astrocytes or morphological changes of astrocytes were noted in samples from postnatal day 11 (P11), P20, or P30. Hence, overexpression of CX3CL1 does not promote astrogenesis or cause astrocytosis. (**B**) Confocal staining of fixed brain samples by SMI22 (green) for GFAP, expressed by astrocytes, showed no obvious changes in proliferation or morphological changes in astrocytes caused by overexpression of CX3CL1, labeled by HA antibody in red. Scale bar is 30 μm. Download Figure 1-2, TIF file

Tg-CX3CL1 mice exhibited no obvious growth defects or reduction in viability. Although the CX3CL1/CX3CR1 pathway has been suggested to modulate microglial migration and activation, enhanced expression of CX3CL1 did not visibly alter either microglia numbers or morphology in young Tg-CX3CL1 mouse brains compared with WT littermates (examples of 2- and 6-month-old samples are shown in Fig. 1-1). Although functional assays or molecular changes remain to be established, marginal activation of microglia might be present in older Tg-CX3CL1 mouse brains (examples of 11- and 20-month-old samples are shown in Fig. 1-1). For astrocytes, no obvious astrocytosis was visibly detected in various stages (early developments in Fig. 1-2*A*, and example of an 11-month-old brain is shown in Fig. 1-2*B*). Instead, the overexpression of CX3CL1 was found to induce neurogenesis, as a visible increase in the thickness of dentate granule cell layers was noted in many Tg-CX3CL1 brains (Fig. 1*C*, examples).

To validate increased neurogenesis, we performed a BrdU pulse-labeling experiment by intraperitoneal injection of BrdU at P21. After a 12 h pulse, we examined the total number of BrdU^+^ cells by immunohistochemistry. It is clear that the total numbers of BrdU^+^ cells were greater in the Tg-CX3CL1 dentate gyrus (DG) when compared with WT littermate controls (Fig. 2*A*). For further validation, we conducted stereological quantification and showed that BrdU^+^ cells in Tg-CX3CL1 SGZ were increased by ∼22.2% compared with those in WT (Fig. 2*B*; 9475.2 ± 912.4 cells per Tg-CX3CL1 DG vs 7464.7 ± 594.7 per WT DG; *N* = 5 animals, **p < 0.01). To analyze neural differentiation, 15 mg/kg BrdU was injected once daily for 5 d beginning at P11, and animals were killed after growing for an additional 4 weeks. Increased neurogenesis was also evident in P45 Tg-CX3CL1 SGZ (Fig. 2*C*). Further quantification validated this increase (Fig. 2*D*).

We also compared BrdU pulse-labeled dividing cells in 8-month-old brains, as neurogenesis was much less active compared with that in early developmental stages. Unlike neurogenesis during early development, significantly fewer BrdU^+^ cells were detectable in WT SGZ (Fig. 2*E*) and even fewer BrdU^+^ cells were found in WT SVZ (Fig. 2*F*), consistent with the fact that adult neurogenesis mainly occurs in the SGZ (Goncalves et al., 2016). Remarkably, increases in BrdU^+^ cells in Tg-CX3CL1 SGZ and SVZ were again evident, showing more active neurogenesis in both neurogenic niches of Tg-CX3CL1 brains (Fig. 2*E*,*F*). Therefore, we demonstrate that enhanced expression of neuronal CX3CL1 induces neurogenesis in the adult mouse brain.

### Overexpressed CX3CL1 activates TGF-β–Smad signaling

**Figure 3.**
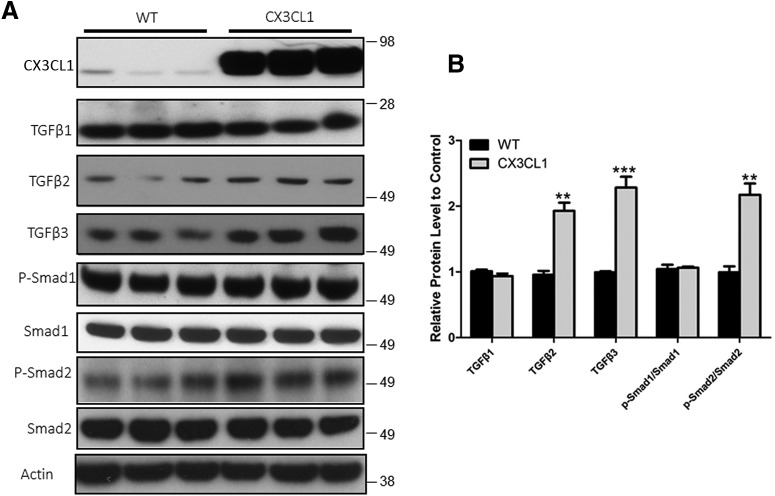
Overexpressed CX3CL1 induces TGF-β2/3–Smad2 signaling. ***A***, Mouse hippocampal lysates (4 months old) were examined by Western blot analyses, and the antibodies used in the assay were specified. ***B***, Bar graphs show normalized comparisons to the loading control (actin). *N* = 3 independent experiments (*p < 0.05, **p < 0.01, ***p < 0.001, Student's *t* test).

We recently showed that membrane-bound CX3CL1 is sequentially cleaved by α-/β- and γ-secretases to release the intracellular domain (CX3CL1-ICD), which can translocate into the nucleus to activate gene expression. Overexpression of CX3CL1-ct leads to increased expression of TGF-β2 and TGF-β3 and the activation of the TGF-β–Smad signaling pathway (Fan et al., 2019). Herein, our Western blot analyses using hippocampal lysates from Tg-CX3CL1 mice again showed higher protein levels of TGF-β3 (Fig. 3*A*). Although overall protein levels of TGF-β2 were less readily detected, quantification showed a significant increase in the Tg-CX3CL1 hippocampus (Fig. 3*B*). Their increased expressions correlated with activation of the downstream molecule Smad2 as levels of phosphorylated Smad2 (p-Smad2) were also clearly increased in Tg-CX3CL1 (Fig. 3*A*,*B*). Noticeably, p-Smad1 and total Smad1 were not significantly different. Hence, in the brain, Smad2 is markedly activated by overexpressed CX3CL1 in neurons, which is consistent with that in Tg-CX3CL1-ct mice.

### Downregulation of Smad2 signaling blocks CX3CL1-mediated neurogenesis

**Figure 4.**
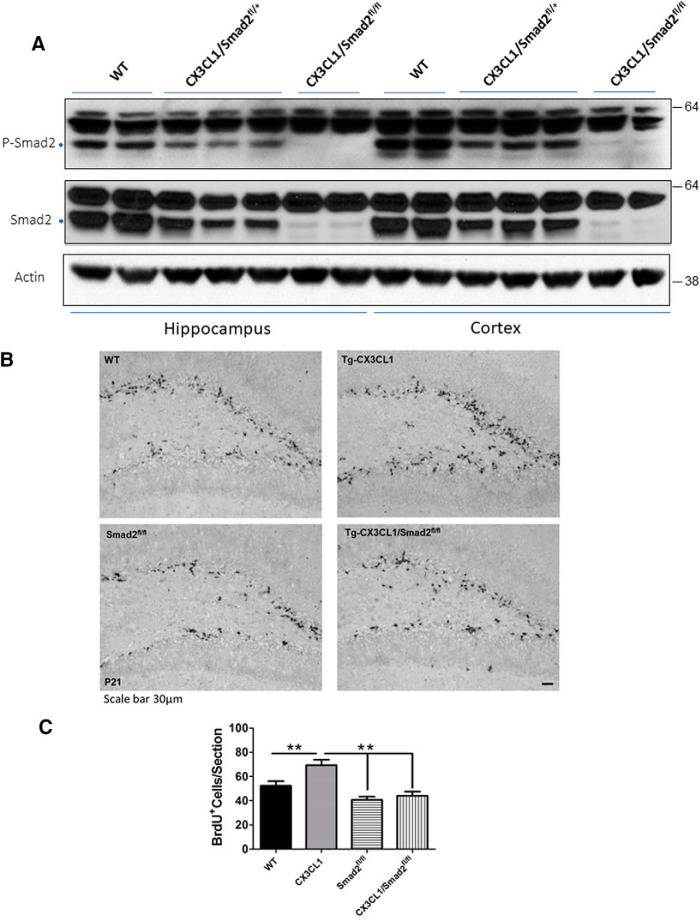
Deletion of Smad2 reverses enhanced neurogenesis in Tg-CX3CL1 mice. ***A***, Deletion of Smad2 in mice was achieved by breeding Smad2^fl/fl^ mice with CamK2a-Cre mice. Hippocampal and cortical protein lysates from the indicated genotypes of mice were examined by Smad2 and phosphorylated with Smad2 antibodies by Western blotting. Reduced expression of Smad2 was seen in CX3CL1/Smad2^fl/+^ mice, and near ablation was observed in CX3CL1/Smad2^fl/fl^ mice. Loading controls are reflected by nonspecific reacted bands and actin antibody. ***B***, P21 mice were pulse labeled by BrdU, and fixed brain sections were examined by immunohistochemical staining with anti-BrdU antibody. Scale bar, 30 μm. ***C***, Quantification was conducted to confirm significance in neurogenesis among genotypes of mice (*N* = 3 animals; *p < 0.05, **p < 0.01, ***p < 0.001, two-way ANOVA with *post hoc* Bonferroni's test).

Smad2-deficient mice were previously shown to be embryonic lethal (Nomura and Li, 1998; Weinstein et al., 1998), and therefore *Smad2^fl/fl^* mice with *loxP* sites flanking exon 2 of Smad2 were later produced (Ju et al., 2006). To determine the contribution of Smad2 to neurogenesis, we bred Tg-CX3CL1 mice with Smad2 conditional deletion mice (https://www.jax.org/strain/022074). We chose CaMKIIα-cre transgenic mice (T29–1; https://www.jax.org/strain/005359) to express Cre recombinase and delete Smad2 in forebrain neurons in CX3CL1/*Smad2^fl/fl^* mice. Our Western blot analyses showed that the expressed Cre recombinase effectively deleted Smad2 in CX3CL1/Smad2^fl/+^ and CX3CL1/Smad2^fl/fl^ mouse neurons as total Smad2 and p-Smad2 levels were correspondingly reduced (Fig. 4*A*). Tg-CX3CL1 with homozygous deletion of Smad2 showed dramatic reduction in Smad2 expression, while the heterozygosity of Smad2 resulted in less reduction in Smad2 and p-Smad2 compared with littermate controls, which expressed no cre in Smad2^fl/fl^ mice.

We then compared neurogenesis using the same approach as described above and found that the deletion of Smad2 in P21 Samd2^fl/fl^ mice by Cre recombinase in the forebrain significantly decreased neurogenesis in their SGZ regions when compared with nonfloxed littermate controls (referred as WT; Fig. 4*B*). While enhanced neurogenesis was consistently shown in Tg-CX3CL1 SGZ, this enhanced neurogenesis was significantly reduced in CX3CL1/Smad2^fl/fl^ mouse SGZ. Further quantification confirmed this significant reduction (Fig. 4*C*; 52.33 ± 3.756 in WT vs 69.33 ± 4.410 in Tg-CX3CL1; 40.67 ± 2.603 in Samd2^fl/fl^ vs 44.00 ± 3.512 in CX3CL1/Smad2^fl/fl^ mouse SGZ; *N* = 3 pairs of mice, *p < 0.05, **p < 0.01). Quantification was conducted to confirm significance in neurogenesis among genotypes of mice (*N* = 3 animals, **p < 0.01, two-way ANOVA with *post hoc* Bonferroni's test). Hence, Smad2 signaling activity contributes to CX3CL1-mediated enhanced neurogenesis.

### Overexpressed CX3CL1 in AD Tau mouse models reverses neuronal loss

Since overexpressed neuronal CX3CL1 enhances neurogenesis, we asked whether this enhancement could reverse neurodegeneration in AD mouse models. It has been reported that PS19 transgenic mice (P301S Tg mice), expressing the P301S mutant form of human microtubule-associated protein tau driven by the Prp promoter, showed a significant loss of neurons in the hippocampus and ventricular dilatation (brain atrophy) starting at between 8 and 9 months of age (Yoshiyama et al., 2007). Yoshiyama et al. (2007) further reported a phenotype of hunched back and paralysis, followed by the inability to intake food by 10 months in this model, with ∼80% of the animals dying by 12 months of age.

**Figure 5.**
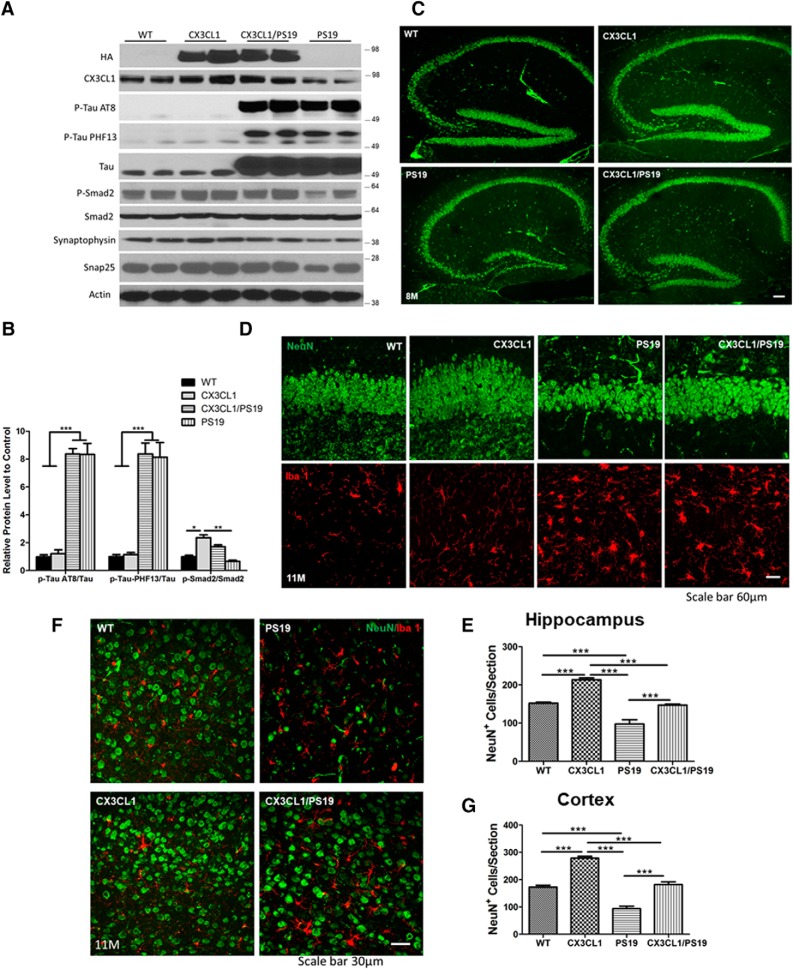
Overexpressed neuronal CX3CL1 rescues neuronal loss in PS19 mice. ***A***, PS19 mice expressing mutant tau (P301S) in neurons. Western blot analyses from hippocampal lysates showed significantly elevated expression of total tau, and this elevation was maintained in Tg-CX3CL1/PS19 mice. Phosphorylated tau, detected by AT8 or PF13 antibodies, showed similar hyperphosphorylation in PS19 and Tg-CX3CL1/PS19 brains. Moreover, AT8^+^ punctae remained in Tg-CX3CL1/PS19 brains (Figure 5-1). CX3CL1 levels were lowered in PS19 brains, but were elevated due to CX3CL1 overexpression validated by two antibodies. p-Smad2 and synaptophysin levels were lower in PS19 mice, and this reduction was reversed by overexpression of CX3CL1. ***B***, Bar graphs showed relative levels of AT8^+^ tau/tau, PHF13^+^ tau/tau, and p-Smad2/Smad2 (*N* = 2 in each group; three experiments; *p < 0.05, **p < 0.01, ***p < 0.001, two-way ANOVA with *post hoc* Bonferroni's test). ***C***, Loss of neurons in CA and DG was visible in PS19 mice, and this reduction was partially rescued. ***D***, Enlarged view of DG showing dramatic loss of neurons labeled by NeuN (green) in PS19 mice. Activation of microglia, labeled by Iba1 antibody, was evident in PS19 mice and also in Tg-CX3CL1/PS19 mice. ***F***, Loss of neurons was visible in the cortical region of PS19 mice, and this reduction was significantly reversed after overexpression of CX3CL1 in Tg-CX3CL1/PS19 mice. Scale bar, 60 μm. ***E***, ***G***, Quantification of neurons in images of hippocampal (***E***) and cortical (***G***) regions, as specified (*N* = 3 slices each; *p < 0.05, **p < 0.01, ***p < 0.001, two-way ANOVA with *post hoc* Bonferroni's test).

We therefore compared PS19 transgenic mice with those expressing CX3CL1 transgene (Tg-CX3CL1/PS19 mice) in a longitudinal study. We noted that neither the expression of tau transgene nor the levels of AT8^+^ or PHF-13^+^ tau was significantly altered by overexpressed CX3CL1 in Tg-CX3CL1/PS19 mice, based on Western analyses (Fig. 5*A*, example of 8-month-old samples, *B*, bar graphs). Smad2 phosphorylation in PS19 transgenic mice was lower compared with WT littermates, but was reversed to higher in Tg-CX3CL1/PS19 mice, although it was not as high as that in Tg-CX3CL1 mice. We also noted that PS19 mice showed a significant reduction in synaptophysin and SNAP25 (Fig. 5*A*), two proteins that are critical for synaptic strength. CX3CL1 overexpression markedly reversed this reduction.

10.1523/JNEUROSCI.1333-19.2019.f5-1 Figure 5-1 **No alteration in tau pathology results from overexpression of CX3CL1**. Fixed brain sections were labeled with AT8 antibody for phosphorylated tau and HA antibody for CX3CL1 transgene. It is clear that overexpressing CX3CL1 in Tg-CX3CL1/PS19 mice did not significantly alter the tau pathology. Scale bar is 30 μm. Download Figure 5-1, TIF file

Mature neurons in 8-month-old mice were labeled with NeuN antibody. At this age, PS19 transgenic mice had already exhibited a significant loss of granule cells in the dentate gyrus compared with WT littermates, while this loss was reversed by overexpressed CX3CL1 (Fig. 5*C*). The loss of neurons in CA regions was more evident in 11-month-old PS19 transgenic mice (Fig. 5*D*,*E*, quantification), consistent with this prior report (Yoshiyama et al., 2007). The most dramatic loss of neurons was seen in cortical regions, as the density of cortical neurons was clearly sparse (Fig. 5*F*,*G*, quantification). Our data showed that increased expression of CX3CL1 was able to reverse this neuronal loss in the two examined age groups.

### Overexpressed CX3CL1 in AD mouse models increases life span and enhances learning and memory

**Figure 6.**
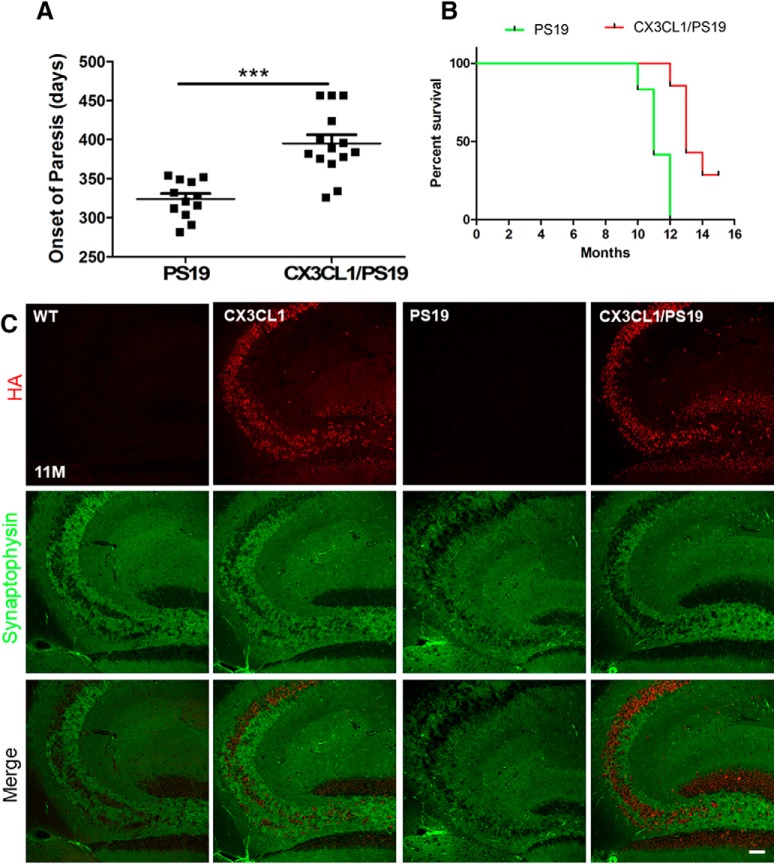
Increased survival times and synaptic densities in Tg-CX3CL1/PS19 mice. ***A***, ***B***, PS19 mice were not able to survival beyond 12 months, and this survival time was significantly increased by overexpression of CX3CL1 in Tg-CX3CL1/PS19 mice. However, Few of the Tg-CX3CL1/PS19 mice lived to 18 months of age, as reflected in the Kaplan–Meier curve (*N* = 14 in the Tg-CX3CL1/PS19 mice in the Tg-CX3CL1/PS19 mice group; ***p < 0.001, Student's *t* test). Both WT and Tg-CX3CL1 mice have normal life spans and easily live beyond 2 years of age. ***C***, Confocal staining was performed to examine synaptic density labeled by synaptophysin in the hippocampus, and PS19 mice showed severely reduced synaptic density. Scale bar, 30 μm.

The life span of PS19 transgenic mice have been previously shown to be shortened (Yoshiyama et al., 2007), and we found that no PS19 transgenic mice were able to survive beyond 12 months of age in our experiments. The occurrence of paralysis in PS19 transgenic cohorts was seen at an average of 321 ± 23 d (Fig. 6*A*,*B*), consistent with the original report (Yoshiyama et al., 2007). Remarkably, a majority of Tg-CX3CL1/PS19 mice survived beyond 12 months with an average age of 397 ± 41 d. We also noted that 3 of these 14 mice (20%) lived up to the age of 18 months (all mice were killed at this age for biochemical analyses). Tg-CX3CL1 mice had a normal life span compared with WT littermates, although it was not examined whether they would have had a longer life span.

Enhanced neurogenesis in Tg-CX3CL1 mouse brains correspondingly increased synaptic density labeled by synaptophysin (Fig. 6*C*), which is consistent with the changes in synaptophysin protein levels shown in Figure 5*A*. On the contrary, synaptic density in PS19 transgenic mouse brains was reduced dramatically, and this reduction was reversed by overexpressed CX3CL1 (Fig. 6*C*).

### Improved cognitive functions by overexpressed CX3CL1

**Figure 7.**
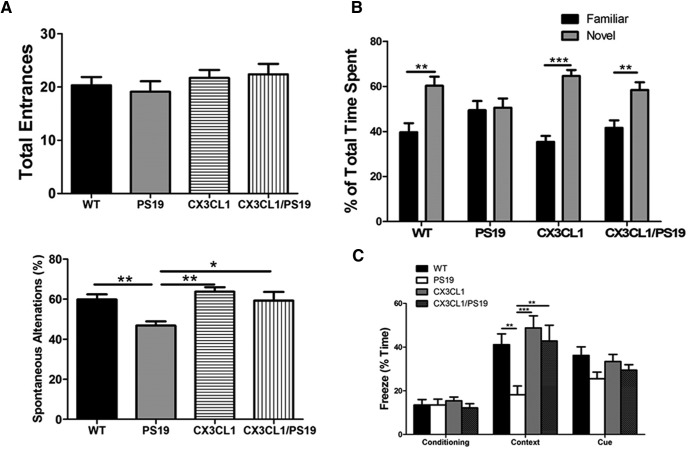
Increased learning and memory in Tg-CX3CL1/PS19 mice. ***A***, A Y-maze test was conducted to assess spatial learning memory, and PS19 mice showed a clear reduction in spontaneous alternations, while this reduction was reversed in Tg-CX3CL1/PS19 mice. Total arm entrances on the Y-maze did not differ among the different genotypes of mice. ***B***, A novel object recognition test was conducted to assess recognition memory. PS19 mice showed no discrimination between novel and familiar objects on the test, and this impairment was reversed by overexpression of CX3CL1 in Tg-CX3CL1/PS19 mice. ***C***, A contextual fear-conditioning test was performed to assess hippocampus-dependent memory in a fear-conditioning chamber. There were no significant differences in the percentage of freeze time during the day 1 conditioning test among different genotypes of mice. Percentage of total freeze time of mice on day 2 was compared with contextual learning ability. PS19 mice exhibited significantly decreased freezing time, suggesting impairment in memory, while mice expressing CX3CL1 exhibited reversal of this impairment. The test of tone-mediated cue memory on day 3 was also reduced in PS19, although it did not reach statistical significance (*N* = 10 in WT and PS19, *N* = 11 in Tg-CX3CL1, and *N* = 13 in Tg-CX3CL1/PS19 groups; *p < 0.05, **p < 0.01, ***p < 0.001, two-way ANOVA with *post hoc* Bonferroni's test for Y-maze experiment, and three-way ANOVA with Tukey's multiple-comparisons test for novel object recognition and contextual fear-conditioning experiments).

To examine the functional changes arising from increased synaptic density by CX3CL1 expression, we conducted learning and memory tests. We first conducted a Y-maze spontaneous alternation test, which measures spatial working memory. While all four genotypes of mice (WT, Tau-transgenic, CX3CL1-transgenic, and CX3CL1/PS19 double transgenic) at the age of 8 months showed a similar number of total entrances into the three arms, PS19 tau transgenic mice displayed significantly fewer spontaneous alternations (Fig. 7*A*; *N* = 12, **p < 0.01, two-way ANOVA). Mice overexpressing CX3CL1 showed a slight increase in total entrances, but no obvious enhancement in working memory despite increased neurogenesis at this age (*N* = 12, *p < 0.05, one-way ANOVA). When CX3CL1 was overexpressed in PS19 Tau transgenic background, reduced working memory was rescued, likely related to reduced neuronal loss (*N* = 12, *p < 0.05, two-way ANOVA).

We then conducted a novel object recognition test, which is used to test for recognition memory (Bevins and Besheer, 2006; Antunes and Biala, 2012). This test measures exploring time spent with a novel object compared with a familiar object. While all four genotypes of mice spent a similar total time exploring objects, PS19 transgenic mice showed no discrimination between novel and familiar objects (Fig. 7*B*). This impairment was remarkably rescued by the overexpression of CX3CL1 in Tg-CX3CL1/PS19 mice (*N* = 12, ***p < 0.001, two-way ANOVA).

We also conducted a contextual fear-conditioning test according to the previously published procedure (Luo et al., 2014). PS19 mice clearly exhibited impaired learning and memory when they were placed back into the conditioned chamber on day 2, with only 18.24 ± 3.948% (*N* = 10) freezing time compared with 41.10 ± 4.935% in WT mice (*N* = 11, Fig. 7*C*). This impaired conditioned fear memory was ameliorated when CX3CL1 was overexpressed in Tg-CX3CL1/PS19 mice (42.77 ± 7.204% in 12 Tg-CX3CL1/PS19 mice vs 48.75 ± 5.553% in 13 Tg-CX3CL1 mice; *p < 0.05, three-way ANOVA).

## Discussion

CX3CL1 is a type I transmembrane cytokine; its N-terminal domain contains a C-XXX-C motif, which is capable of binding to CX3CR1 to exert signaling transduction between neurons and microglia. Under normal conditions, CX3CL1 is mainly expressed by neurons and at a low levels by astrocytes, but not microglia; CX3CR1 is predominantly expressed by microglia (Hatori et al., 2002; Hughes et al., 2002; Hulshof et al., 2003). In our initial study of mice overexpressing neuronal CX3CL1 (Tg-CX3CL1), we found that changes of microglia were not readily visible in young Tg-CX3CL1 mouse brains (Fig. 1-1). Further studies are required to confirm potential molecular and functional changes in microglia when CX3CL1 is overexpressed. It will be more informative when mice overexpressing only C-terminal CX3CL1 will be examined in parallel.

Activation of microglia is evident in PS19 transgenic mice, clearly arising from abnormal tau phosphorylation (Fig. 5*C*,*D*, microglia morphology). It appears that microglial activation in Tg-CX3CL1/PS19 mice was comparable to that in PS19 mice. This is consistent with the fact that total levels of AT8 or PHF13 on the Western blot were not visibly altered by overexpressed CX3CL1. Confocal examination of brain sections with antibody AT8 also showed that strong AT8 immunity of neurons in PS19 transgenic and Tg-CX3CL1/PS19 mouse brains were comparable, and tau aggregations were also noted in Tg-CX3CL1/PS19 brains (Fig. 5-1); a nonspecific background such as blood vessels was detected in WT brains and Tg-CX3CL1. Intriguingly, one prior study expressing soluble CX3CL1 via adenoviral transformation in a Tg4510 model of tauopathy was shown to reduce tau pathology (Nash et al., 2013), and CX3CR1 deficiency to impair microglia to internalize tau aggregates (Bolós et al., 2017). Glia cells contribute to the reduction of AD pathology including removal of tau aggregates (Leyns and Holtzman, 2017; Wang et al., 2018; Vogels et al., 2019). Although enhanced expression of neuronal CX3CL1 in the PS19 transgenic background has no obvious effect on reducing tau pathology, it prevents or reduces neurodegeneration. It should be noted that multiple effects existed in our study, which may differ from the other two studies, including the differences in mouse models and the full-length versus the soluble form of CX3CL1 as well as the C-terminal back signaling (Fan et al., 2019).

Enhanced neurogenesis in Tg-CX3CL1 mice begins during early development and lasts throughout their life span, consistent with that seen in Tg-CX3CL1-ct mice. This enhanced neurogenesis occurs not only in the SGZ, which is the predominant region for adult neurogenesis, but also in the SVZ of the lateral ventricle. A prior study conducted by infusive expression of soluble CX3CL1 was also found to enhance hippocampal neurogenesis, and this effect was blocked by the infusion of neutralizing antibody against CX3CR1 (Bachstetter et al., 2011). It also suggests that the loss of CX3CR1 function impairs hippocampal neurogenesis. In this study, we cannot exclude the contribution of overexpressed CX3CL1 in Tg-CX3CL1 mice to enhanced neurogenesis through the CX3CL1-CX3CR1 effect, but we have focused our attention on the role of CX3CL1-ct back signaling, mainly because Tg-CX3CL1-ct mice have similarly enhanced neurogenesis in SGZ and SVZ (Fan et al., 2019).

We demonstrate that mice expressing CX3CL1-ct exhibit increased expression of TGF-β2 and TGF-β3, but not TGF-β1. TGF-β2/3 evokes its physiological functions through binding to two different receptor serine/threonine kinases, type I and type II TGF-β receptors (Shi and Massagué, 2003). Binding of TGF-β2/3 to the receptor, which is a tetrameric complex, will trigger recruitment of the anchor protein SARA (Smad anchor for receptor activation) and subsequently will increase phosphorylation of the cytoplasmic signal transducers Smad2 and Smad3. Phosphorylated Smad2 and Smad3 will translocate into the nucleus, where they regulate gene expression. We showed that mice overexpressing either CX3CL1 (Fig. 3 in this study) or CX3CL1-ct (Fan et al., 2019) similarly elevate the expression of TGF-β2 and TGF-β3 as well as the activation of Smad2 and Smad3, indicating that this effect is CX3CR1 independent.

Smad2 and Smad3 appear to control gene expression in a combination of homodimeric and heterodimeric states, and they display differential roles in embryonic development. Complete deletion of the *Smad2* gene in mice causes embryonic lethality due to a failure to establish an anterior–posterior axis of gastrulation and mesoderm formation (Weinstein et al., 2000), while mice deficient in the *Smad3* gene are viable and can survive for several months (Datto et al., 1999; Yang et al., 1999), indicating the existence of differential roles *in vivo*. To determine whether Smad2 has a role in neurogenesis, we obtained a mouse model with conditional deletion of Smad2 (Liu et al., 2004). Mice with Smad2 deleted from their forebrain neurons showed a small reduction in adult neurogenesis in our study, and this reduction could reverse the enhanced neurogenesis in Tg-CX3CL1 mice (Fig. 4), indicating that TGF-β2/3–Smad2 pathway contributes to the enhanced neurogenesis by CX3CL1 ICD.

The observed neurogenesis in Tg-CX3CL1 mice prompted us to test whether enhanced adult neurogenesis would be beneficial for reversing neuronal loss in neurodegenerative diseases such as Alzheimer's disease. A recent study shows exacerbated cognitive deficits in AD by disrupted adult neurogenesis (Hollands et al., 2017). We chose PS19 mice for this purpose as this mouse model displays neuronal loss in broad brain regions and the degrees of neuronal loss and brain atrophy correlate with severely reduced survival time, with few PS19 mice living beyond 12 months of age (Yoshiyama et al., 2007). As demonstrated in Figure 6*A*, overexpressed CX3CL1 in the PS19 mouse model significantly increased the life span. Most of the Tg-CX3CL1/PS19 mice, except two, lived beyond 12 months of age, with three eventually killed at the age of 18 months for analyses. This increase can likely be attributed to the reversal of neuronal loss, as newborn neurons may replenish degenerated neurons to enhance synaptic connectivity. We noted that synaptic density was significantly greater in the Tg-CX3CL1/PS19 than in the PS19 hippocampal region (Fig. 6*B*). Consequently, cognitive function, measured by performances on Y-maze, open field, and contextual fear-conditioning tests, was significantly improved in Tg-CX3CL1/PS19 mice compared with PS19 mice.

In summary, we demonstrate a role of CX3CL1 in promoting adult neurogenesis in broad brain regions (SGZ and SVZ), through activating the TGF-β–Smad2 signaling pathway. This effect is attributable to the C-terminal fragment, which can induce expression of the TGF-β2/3–Smad2 pathway. Since hippocampal neurogenesis persists in the aged and diseased human brain (Tobin et al., 2019), enhancing neuronal CX3CL1, mainly the C-terminal fragment, may well be a viable therapeutic strategy for blocking or reversing neuronal loss in the treatment of patients with Alzheimer's disease or related neurodegenerative diseases.

## References

[B1] Antunes M, Biala G (2012) The novel object recognition memory: neurobiology, test procedure, and its modifications. Cogn Process 13:93–110. 10.1007/s10339-011-0430-z 22160349 PMC3332351

[B2] Bachstetter AD, Morganti JM, Jernberg J, Schlunk A, Mitchell SH, Brewster KW, Hudson CE, Cole MJ, Harrison JK, Bickford PC, Gemma C (2011) Fractalkine and CX 3 CR1 regulate hippocampal neurogenesis in adult and aged rats. Neurobiol Aging 32:2030–2044. 10.1016/j.neurobiolaging.2009.11.022 20018408 PMC2889032

[B3] Bazan JF, Bacon KB, Hardiman G, Wang W, Soo K, Rossi D, Greaves DR, Zlotnik A, Schall TJ (1997) A new class of membrane-bound chemokine with a CX3C motif. Nature 385:640–644. 10.1038/385640a0 9024663

[B4] Bevins RA, Besheer J (2006) Object recognition in rats and mice: a one-trial non-matching-to-sample learning task to study “recognition memory”. Nat Protoc 1:1306–1311. 10.1038/nprot.2006.205 17406415

[B5] Bhaskar K, Konerth M, Kokiko-Cochran ON, Cardona A, Ransohoff RM, Lamb BT (2010) Regulation of tau pathology by the microglial fractalkine receptor. Neuron 68:19–31. 10.1016/j.neuron.2010.08.023 20920788 PMC2950825

[B6] Bolós M, Llorens-Martín M, Perea JR, Jurado-Arjona J, Rábano A, Hernández F, Avila J (2017) Absence of CX3CR1 impairs the internalization of tau by microglia. Mol Neurodegener 12:59. 10.1186/s13024-017-0200-1 28810892 PMC5558740

[B7] Borchelt DR, Ratovitski T, van Lare J, Lee MK, Gonzales V, Jenkins NA, Copeland NG, Price DL, Sisodia SS (1997) Accelerated amyloid deposition in the brains of transgenic mice coexpressing mutant presenilin 1 and amyloid precursor proteins. Neuron 19:939–945. 10.1016/S0896-6273(00)80974-5 9354339

[B8] Braak H, Braak E (1997) Diagnostic criteria for neuropathologic assessment of Alzheimer's disease. Neurobiol Aging 18:S85–S88. 10.1016/S0197-4580(97)00062-6 9330992

[B9] Cardona AE, Pioro EP, Sasse ME, Kostenko V, Cardona SM, Dijkstra IM, Huang D, Kidd G, Dombrowski S, Dutta R, Lee JC, Cook DN, Jung S, Lira SA, Littman DR, Ransohoff RM (2006) Control of microglial neurotoxicity by the fractalkine receptor. Nat Neurosci 9:917–924. 10.1038/nn1715 16732273

[B10] Cho SH, Sun B, Zhou Y, Kauppinen TM, Halabisky B, Wes P, Ransohoff RM, Gan L (2011) CX3CR1 protein signaling modulates microglial activation and protects against plaque-independent cognitive deficits in a mouse model of alzheimer disease. J Biol Chem 286:32713–32722. 10.1074/jbc.M111.254268 21771791 PMC3173153

[B11] Datto MB, Frederick JP, Pan L, Borton AJ, Zhuang Y, Wang XF (1999) Targeted disruption of Smad3 reveals an essential role in transforming growth factor beta-mediated signal transduction. Mol Cell Biol 19:2495–2504. 10.1128/MCB.19.4.2495 10082515 PMC84042

[B12] Fan Q, Gayen M, Singh N, Gao F, He W, Hu X, Tsai LH, Yan R (2019) The intracellular domain of CX3CL1 regulates adult neurogenesis and Alzheimer's amyloid pathology. J Exp Med 216:1891–1903. 10.1084/jem.20182238 31209068 PMC6683996

[B13] Fong AM, Erickson HP, Zachariah JP, Poon S, Schamberg NJ, Imai T, Patel DD (2000) Ultrastructure and function of the fractalkine mucin domain in CX(3)C chemokine domain presentation. J Biol Chem 275:3781–3786. 10.1074/jbc.275.6.3781 10660527

[B14] Fuhrmann M, Bittner T, Jung CK, Burgold S, Page RM, Mitteregger G, Haass C, LaFerla FM, Kretzschmar H, Herms J (2010) Microglial Cx3cr1 knockout prevents neuron loss in a mouse model of Alzheimer's disease. Nat Neurosci 13:411–413. 10.1038/nn.2511 20305648 PMC4072212

[B15] Goncalves JT, Schafer ST, Gage FH (2016) Adult neurogenesis in the hippocampus: From stem cells to behavior. Cell 167:897–914. 27814520 10.1016/j.cell.2016.10.021

[B16] Guedes JR, Lao T, Cardoso AL, El Khoury J (2018) Roles of microglial and monocyte chemokines and their receptors in regulating Alzheimer's disease-associated amyloid-beta and tau pathologies. Front Neurol 9:549. 10.3389/fneur.2018.00549 30158892 PMC6104478

[B17] Hatori K, Nagai A, Heisel R, Ryu JK, Kim SU (2002) Fractalkine and fractalkine receptors in human neurons and glial cells. J Neurosci Res 69:418–426. 10.1002/jnr.10304 12125082

[B18] Hollands C, Tobin MK, Hsu M, Musaraca K, Yu TS, Mishra R, Kernie SG, Lazarov O (2017) Depletion of adult neurogenesis exacerbates cognitive deficits in Alzheimer's disease by compromising hippocampal inhibition. Mol Neurodegener 12:64. 10.1186/s13024-017-0207-7 28886753 PMC5591545

[B19] Hu X, He W, Luo X, Tsubota KE, Yan R (2013) BACE1 regulates hippocampal astrogenesis via the Jagged1-notch pathway. Cell Rep 4:40–49. 10.1016/j.celrep.2013.06.005 23831026 PMC3740554

[B20] Hu X, Das B, Hou H, He W, Yan R (2018) BACE1 deletion in the adult mouse reverses preformed amyloid deposition and improves cognitive functions. J Exp Med 215:927–940. 10.1084/jem.20171831 29444819 PMC5839766

[B21] Hughes PM, Botham MS, Frentzel S, Mir A, Perry VH (2002) Expression of fractalkine (CX3CL1) and its receptor, CX3CR1, during acute and chronic inflammation in the rodent CNS. Glia 37:314–327. 10.1002/glia.10037 11870871

[B22] Hulshof S, van Haastert ES, Kuipers HF, van den Elsen PJ, De Groot CJ, van der Valk P, Ravid R, Biber K (2003) CX3CL1 and CX3CR1 expression in human brain tissue: noninflammatory control versus multiple sclerosis. J Neuropathol Exp Neurol 62:899–907. 10.1093/jnen/62.9.899 14533779

[B23] Imai T, Hieshima K, Haskell C, Baba M, Nagira M, Nishimura M, Kakizaki M, Takagi S, Nomiyama H, Schall TJ, Yoshie O (1997) Identification and molecular characterization of fractalkine receptor CX3CR1, which mediates both leukocyte migration and adhesion. Cell 91:521–530. 10.1016/S0092-8674(00)80438-9 9390561

[B24] Ju W, Ogawa A, Heyer J, Nierhof D, Yu L, Kucherlapati R, Shafritz DA, Böttinger EP (2006) Deletion of Smad2 in mouse liver reveals novel functions in hepatocyte growth and differentiation. Mol Cell Biol 26:654–667. 10.1128/MCB.26.2.654-667.2006 16382155 PMC1346892

[B25] Lauro C, Catalano M, Trettel F, Limatola C (2015) Fractalkine in the nervous system: neuroprotective or neurotoxic molecule? Ann NY Acad Sci 1351:141–148. 10.1111/nyas.12805 26084002

[B26] Lee S, Varvel NH, Konerth ME, Xu G, Cardona AE, Ransohoff RM, Lamb BT (2010) CX3CR1 deficiency alters microglial activation and reduces beta-amyloid deposition in two Alzheimer's disease mouse models. Am J Pathol 177:2549–2562. 10.2353/ajpath.2010.100265 20864679 PMC2966811

[B27] Lee S, Xu G, Jay TR, Bhatta S, Kim KW, Jung S, Landreth GE, Ransohoff RM, Lamb BT (2014) Opposing effects of membrane-anchored CX3CL1 on amyloid and tau pathologies via the p38 MAPK pathway. J Neurosci 34:12538–12546. 10.1523/JNEUROSCI.0853-14.2014 25209291 PMC4160782

[B28] Leyns CEG, Holtzman DM (2017) Glial contributions to neurodegeneration in tauopathies. Mol Neurodegener 12:50. 10.1186/s13024-017-0192-x 28662669 PMC5492997

[B29] Limatola C, Ransohoff RM (2014) Modulating neurotoxicity through CX3CL1/CX3CR1 signaling. Front Cell Neurosci 8:229. 10.3389/fncel.2014.00229 25152714 PMC4126442

[B30] Liu Y, Festing MH, Hester M, Thompson JC, Weinstein M (2004) Generation of novel conditional and hypomorphic alleles of the Smad2 gene. Genesis 40:118–123. 10.1002/gene.20072 15452874

[B31] Luo X, He W, Hu X, Yan R (2014) Reversible overexpression of bace1-cleaved neuregulin-1 N-terminal fragment induces schizophrenia-like phenotypes in mice. Biol Psychiatry 76:120–127. 10.1016/j.biopsych.2013.09.026 24210810 PMC3976896

[B32] Nash KR, Lee DC, Hunt JB Jr, Morganti JM, Selenica ML, Moran P, Reid P, Brownlow M, Guang-Yu Yang C, Savalia M, Gemma C, Bickford PC, Gordon MN, Morgan D (2013) Fractalkine overexpression suppresses tau pathology in a mouse model of tauopathy. Neurobiol Aging 34:1540–1548. 10.1016/j.neurobiolaging.2012.12.011 23332170 PMC8970215

[B33] Nomura M, Li E (1998) Smad2 role in mesoderm formation, left-right patterning and craniofacial development. Nature 393:786–790. 10.1038/31693 9655392

[B34] Pan Y, Lloyd C, Zhou H, Dolich S, Deeds J, Gonzalo JA, Vath J, Gosselin M, Ma J, Dussault B, Woolf E, Alperin G, Culpepper J, Gutierrez-Ramos JC, Gearing D (1997) Neurotactin, a membrane-anchored chemokine upregulated in brain inflammation. Nature 387:611–617. 10.1038/42491 9177350

[B35] Paolicelli RC, Bisht K, Tremblay MÈ (2014) Fractalkine regulation of microglial physiology and consequences on the brain and behavior. Front Cell Neurosci 8:129. 10.3389/fncel.2014.00129 24860431 PMC4026677

[B36] Shi Y, Massagué J (2003) Mechanisms of TGF-beta signaling from cell membrane to the nucleus. Cell 113:685–700. 10.1016/S0092-8674(03)00432-X 12809600

[B37] Tobin MK, Musaraca K, Disouky A, Shetti A, Bheri A, Honer WG, Kim N, Dawe RJ, Bennett DA, Arfanakis K, Lazarov O (2019) Human hippocampal neurogenesis persists in aged adults and Alzheimer's disease patients. Cell Stem Cell 24:974–982.e3. 31130513 10.1016/j.stem.2019.05.003 PMC6608595

[B38] Vogels T, Murgoci AN, Hromádka T (2019) Intersection of pathological tau and microglia at the synapse. Acta Neuropathol Commun 7:109. 10.1186/s40478-019-0754-y 31277708 PMC6612163

[B39] Wang H, Li Y, Ryder JW, Hole JT, Ebert PJ, Airey DC, Qian HR, Logsdon B, Fisher A, Ahmed Z, Murray TK, Cavallini A, Bose S, Eastwood BJ, Collier DA, Dage JL, Miller BB, Merchant KM, O'Neill MJ, Demattos RB (2018) Genome-wide RNAseq study of the molecular mechanisms underlying microglia activation in response to pathological tau perturbation in the rTg4510 tau transgenic animal model. Mol Neurodegener 13:65. 10.1186/s13024-018-0296-y 30558641 PMC6296031

[B40] Weinstein M, Yang X, Li C, Xu X, Gotay J, Deng CX (1998) Failure of egg cylinder elongation and mesoderm induction in mouse embryos lacking the tumor suppressor smad2. Proc Natl Acad Sci U S A 95:9378–9383. 10.1073/pnas.95.16.9378 9689088 PMC21346

[B41] Weinstein M, Yang X, Deng C (2000) Functions of mammalian smad genes as revealed by targeted gene disruption in mice. Cytokine Growth Factor Rev 11:49–58. 10.1016/S1359-6101(99)00028-3 10708952

[B42] Yang X, Letterio JJ, Lechleider RJ, Chen L, Hayman R, Gu H, Roberts AB, Deng C (1999) Targeted disruption of SMAD3 results in impaired mucosal immunity and diminished T cell responsiveness to TGF-beta. EMBO J 18:1280–1291. 10.1093/emboj/18.5.1280 10064594 PMC1171218

[B43] Yoshiyama Y, Higuchi M, Zhang B, Huang SM, Iwata N, Saido TC, Maeda J, Suhara T, Trojanowski JQ, Lee VM (2007) Synapse loss and microglial activation precede tangles in a P301S tauopathy mouse model. Neuron 53:337–351. 10.1016/j.neuron.2007.01.010 17270732

