# Supplementary figures and images for: Activated CX3CL1/Smad2 Signals Prevent Neuronal Loss and Alzheimer's Tau Pathology-Mediated Cognitive Dysfunction
Source: J Neurosci. 2020 Jan 29;40(5):1133–44. doi: 10.1523/JNEUROSCI.1333-19.2019 (PMC6989010; doi:10.1523/JNEUROSCI.1333-19.2019)

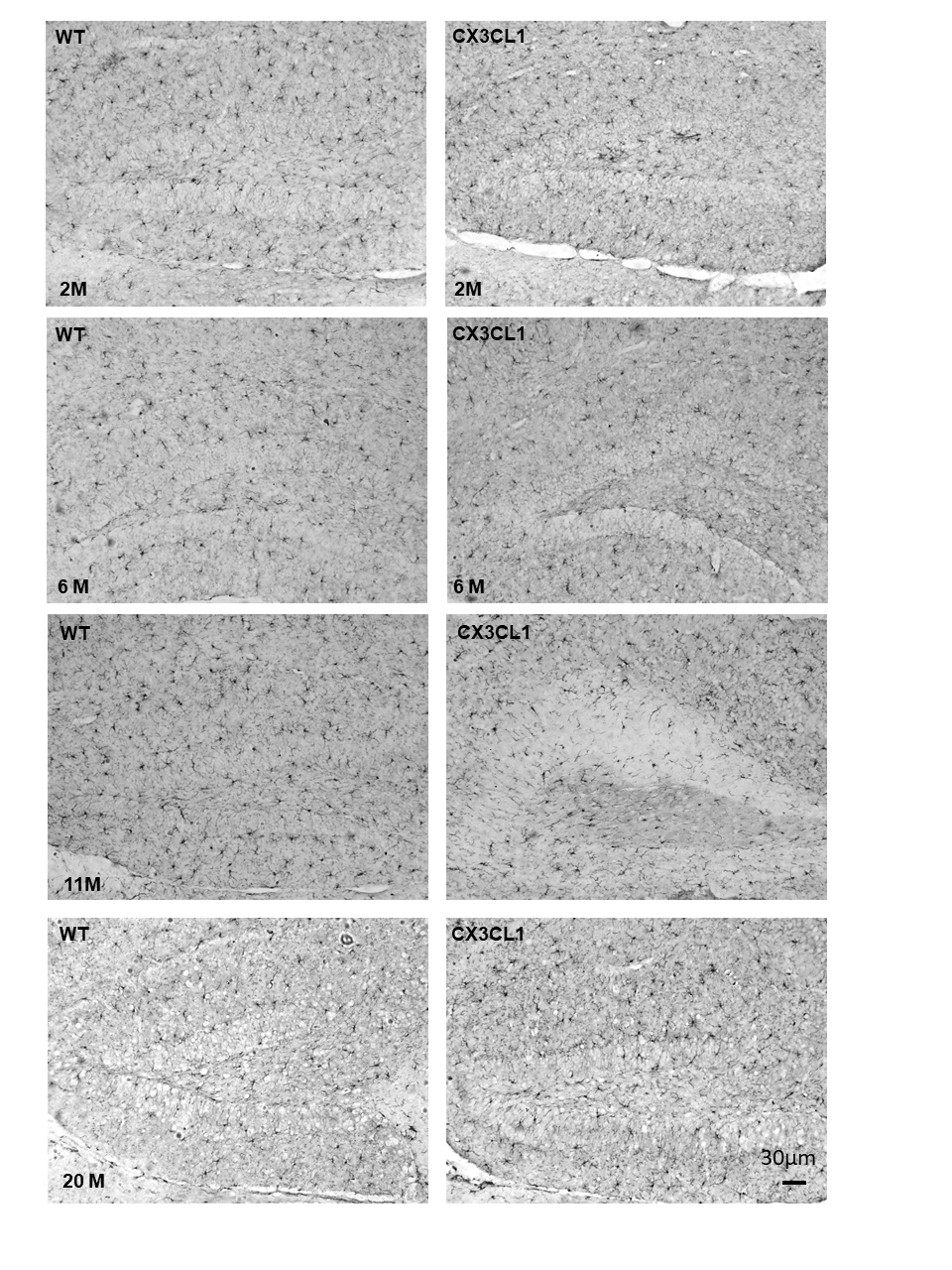

Supplement: Figure 1-1 [file sup_ns-JN-RM-1333-19-s01.tif]

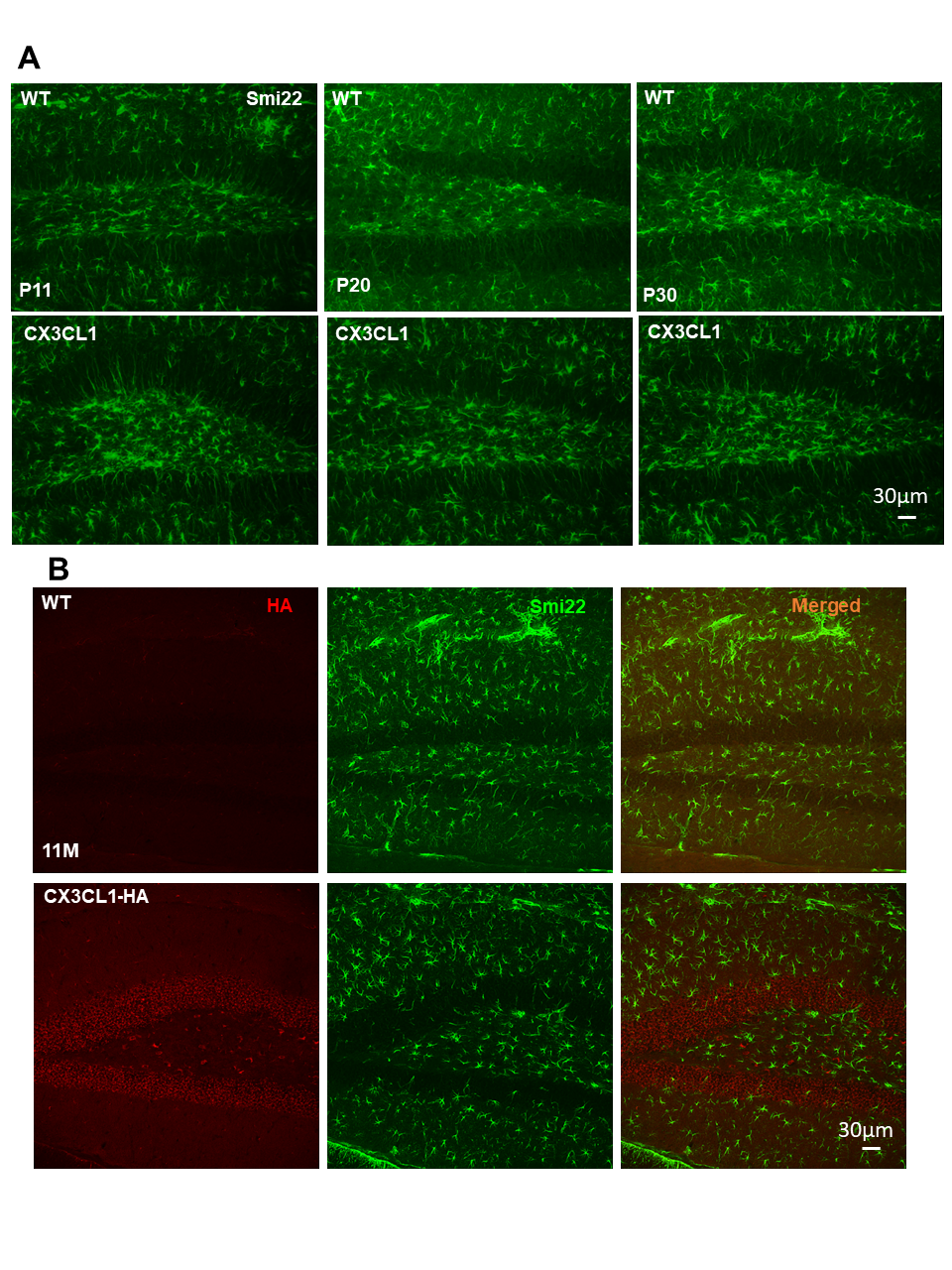

Supplement: Figure 1-2 [file sup_ns-JN-RM-1333-19-s02.tif]

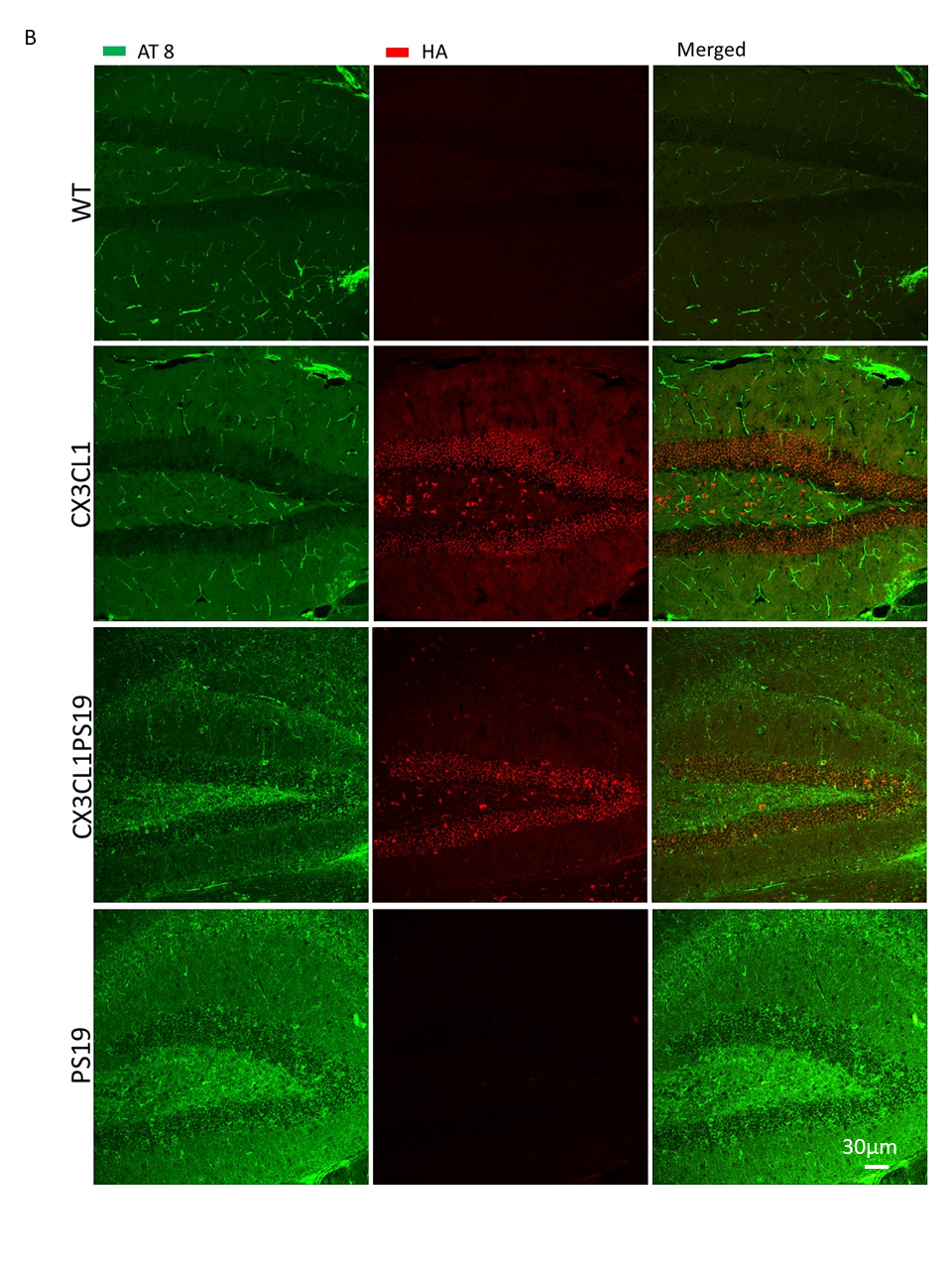

Supplement: Figure 5-1 [file sup_ns-JN-RM-1333-19-s03.tif]
